# Industrial Welfare

**Published:** 1920-05-01

**Authors:** 


### INDUSTRIAL WELFARE.

### Organisations in the Factories.

"The Welfare Worker in Industry" was the subject of an interesting discussion at the London Chamber of Commerce, which was attended by Prince Albert. Some facts were there given, notably by Sir Robert Hadfield, concerning the activities of the Industrial Welfare Society which are worthy of note.

The work started first of all amongst boys, whose interests were watched by a supervisor, but in most cases the men, seeing the usefulness of the supervisor's presence, frequently sought his help. In many firms he acts as secretary of various committees dealing with questions of sanitation, workshop conditions, "safety first," and recreation, and

THE PUBLIC WELL-BEING—(*continued*).

is the focus of the men's activities. Sir Robert Hadfield gave an instance of one large steelworks whose Industrial Welfare Society had nearly 7,000 members, and was carried on on a self-supporting basis by a voluntary levy of 2d. per week. It had its own benefit society, social fixtures, competitions, canteen, and so forth, all under the guidance of the supervisor. Many shipyards have active societies, and great benefits have accrued to firms, to works, and to the community.

Prince Albert expressed his deep interest in the work and aims of the Industrial Welfare Society —of which he is president—and spoke of its extreme importance on the human side of industry in providing opportunities for recreation and other interests, during leisure hours. It was pointed out during the course of the discussion that many new branch offices have been opened, and inquiries about the Society's work have even come from other countries. Next year it is hoped to develop the work amongst women workers, and to appoint representatives in every industrial area. A word of blessing came from a representative of the Amalgamated Society of Engineers who was present at the meeting.

Conditions to be faced.

It is a most vital need that welfare work should be developed in factories, for as the report on Army medical examination shows—a report to which Prince Albert made reference—industrial conditions have a close bearing on national physique, and everything that can be done to counteract the ill-effects is all to the good. Lord Astor and Sir George Newman both alluded to this subject indirectly last week. The question of the great number of defective school children is not altogether unconnected with industrial circumstances, though that is only part of the trouble. What is more to the point perhaps is that the expenditure in cash benefits for sickness and disablement under the Insurance Act reveals the serious fact that there are annually over 14,000,000 weeks of industrial time lost through sickness or disablement. Much of this sickness and disablement is preventable and reducible; the contributory factors include venereal disease, alcoholism, tuberculosis, and slums. Similar considerations arise in the case of maternity, which is a normal function and not a disease, and we should try to reduce the death and disablement caused by and associated with childbirth. In industry a large number of women are periodically diverted from the production of cash capital to the production of human capital. If mothers got proper pre-natal, natal, and post-natal care there would not be 15,000 babies dying every year within one week of birth. These things are all being better realised nowadays, and the forthcoming Health Week should be able to achieve much in bringing still further enlightenment regarding existing facts, and in developing a public conscience which will insist upon all possible remedies.

